# Novel Therapeutic Horizons: *SNCA* Targeting in Parkinson’s Disease

**DOI:** 10.3390/biom14080949

**Published:** 2024-08-06

**Authors:** Alessio Maria Caramiello, Valentina Pirota

**Affiliations:** Department of Chemistry, University of Pavia, Viale Taramelli 10, 27100 Pavia, Italy; alessiomaria.caramiello@unipv.it

**Keywords:** alpha-synuclein, Parkinson’s disease, *SNCA* gene, gene therapy, ASOs, G-quadruplex

## Abstract

Alpha-synuclein (αSyn) aggregates are the primary component of Lewy bodies, which are pathological hallmarks of Parkinson’s disease (PD). The toxicity of αSyn seems to increase with its elevated expression during injury, suggesting that therapeutic approaches focused on reducing αSyn burden in neurons could be beneficial. Additionally, studies have shown higher levels of *SNCA* mRNA in the midbrain tissues and substantia nigra dopaminergic neurons of sporadic PD post-mortem brains compared to controls. Therefore, the regulation of *SNCA* expression and inhibition of αSyn synthesis could play an important role in the pathogenesis of injury, resulting in an effective treatment approach for PD. In this context, we summarized the most recent and innovative strategies proposed that exploit the targeting of *SNCA* to regulate translation and efficiently knock down cytoplasmatic levels of αSyn. Significant progress has been made in developing antisense technologies for treating PD in recent years, with a focus on antisense oligonucleotides and short-interfering RNAs, which achieve high specificity towards the desired target. To provide a more exhaustive picture of this research field, we also reported less common but highly innovative strategies, including small molecules, designed to specifically bind 5′-untranslated regions and, targeting secondary nucleic acid structures present in the *SNCA* gene, whose formation can be modulated, acting as a transcription and translation control. To fully describe the efficiency of the reported strategies, the effect of αSyn reduction on cellular viability and dopamine homeostasis was also considered.

## 1. Introduction

Parkinson’s disease (PD) is the second most common neurodegenerative disorder, affecting more than 8.5 million individuals worldwide, a figure that is expected to reach over 12 million by 2040 (https://www.parkinson.org/ (accessed on 26 September 2023)). It is an age-related degenerative brain condition that typically develops beyond the age of 60. However, 10–15% of cases are diagnosed at a younger age (<40) [1]. PD is 1.5 times more common in males than in females [2]. It is a movement disorder caused by a growing and selective degeneration of dopaminergic neurons in the substantia nigra pars compacta of the midbrain and other monoaminergic neurons in the brain stem [3]. These areas of the central nervous system are mainly related to the control of voluntary movements, so much so that the main symptoms refer to the motor sphere, including slowness of movement, resting tremors, stiffness at rest, and postural instability. In the more advanced stages of the disease, other non-motor symptoms develop like changes in speech and voice, difficulty in swallowing, memory loss, anxiety, and depression [4]. Associated with the large social impact of the disease, there is also the absence of curative treatments. The current mainstay treatment for PD remains L-dopa, the dopamine precursor, able to improve motor symptoms. As for L-dopa, the few new compounds commercialized for PD treatment have only symptomatic effects [5]. As a result, their administration may not prevent the spreading and progression of PD and may be associated with many side effects [6]. This makes PD the fastest-growing neurological disorder in terms of prevalence, impairment, and death.

The majority of idiopathic PD cases represent a late-onset sporadic form whose neuropathological hallmarks are Lewy bodies, spherical filamentous protein inclusions found in the cytoplasm of neurons, often near the nucleus, and enriched in aggregated α-synuclein (αSyn) protein and ubiquitin [7]. αSyn is a presynaptic protein of 140 residues, encoded by the *SNCA* gene, composed of the following three different domains: an N-terminal region, which encompasses residues 1–60, the highly hydrophobic self-aggregating sequence known as non-amyloid-component, which initiates fibrillization, constituted by residues 61–95, and the acidic C-terminal region, residues 96–140, which is essential for blocking rapid αSyn filament assembly [8]. The physiological role of αSyn has been related to membrane binding, synaptic vesicle recycling, and dopamine (DA) metabolism [9]. This 140-amino-acid polypeptide appears to interact with proteins that regulate DA synthesis and uptake by altering the activity of tyrosine hydroxylase (TH), a key enzyme in DA synthesis, and inhibiting aromatic amino acid decarboxylase, which regulates the conversion of Levodopa (L-dopa) into DA [10]. In this regard, αSyn neurotoxicity seems to be DA-dependent, as it causes apoptosis in cultured human dopaminergic neurons but protects non-dopaminergic human cortical neurons. It has also been proposed that intracellular αSyn may be associated with plasma and vesicular membranes [11]. The misfolding of αSyn can reduce its ability to bind to membranes, resulting in its accumulation in the cytosol and subsequent oligomerization and aggregation in vivo. This process involves the formation of β-sheet-rich pre-fibrillar aggregates, which leads to the formation of pores in membranes, leading to increased permeability, calcium influx, and, ultimately, cell death [12]. Although the mechanism of the formation of Lewy bodies and their specific impact on dopaminergic neurons in the substantia nigra (SN) occurring in the disease remains unclear, there is growing evidence that disruptions in metal homeostasis and oxidative stress play critical roles in various age-related neurodegenerative diseases, including PD [13,14,15]. In particular, phosphorylated αSyn at Serine-129 residues (pSer129) is recognized as the predominant protein form found in Lewy bodies aggregates; while only 4% of αSyn is phosphorylated in healthy brains, the percentage reaches 90% in brains with Lewy pathology [16]. In this context, the discovery of the oligomerization of αSyn during injury has paved the way for new therapeutic strategies aimed at decreasing its synthesis or increasing its clearance, preventing and/or removing its toxic deposits [4]. Many different therapeutic approaches have been investigated in the last decade and, among them, the modulation of *SNCA* gene expression has attracted increasing interest. The involvement of *SNCA* deregulation in PD pathogenesis also derives from the evidence that *SNCA* locus multiplication (duplication or triplication) [17] or *SNCA* gene mutations are strongly linked to severity in familial parkinsonism, showing dosage-dependent toxicity [3].

Therefore, it is convincing that the downregulation of transcriptional and post-transcriptional *SNCA* mRNA processing can reduce all forms of αSyn protein, positively contributing to PD pathogenesis and treatments.

Herein, we reported a summary of the most recent and pioneering strategies that have exploited the targeting of the *SNCA* gene to balance the available amount of αSyn by preventing protein spread and aggregation.

## 2. Antisense Strategy

Among all the molecular approaches used in medicinal chemistry, the use of antisense oligonucleotides (ASOs) is undoubtedly the most specific and rapid approach to downregulating the expression of a target gene via the recognition of cellular RNAs. ASOs are synthetic, short (13–25 nucleotides), single-stranded deoxynucleotide or ribonucleotide analogs, chosen to be uniquely complementary, at least in principle, to the mRNA target structure of interest [18]. Usually, a downregulation in the translation of the target gene is induced with the recruitment of Ribonuclease H, which degrades the RNA strand of an RNA–DNA heteroduplex, reducing the levels of the target mRNA. Other mechanisms are also possible, one being translation inhibition through a physical blockage of the ribosomal machinery, preventing the translation of the target mRNA into protein, and another being the splicing modulation, when ASOs bind to splice sites or splicing enhancers/silencers thus altering pre-mRNA splicing patterns [19,20]. During the last decade, the use of ASOs as the most selective tool in the targeting of RNA has been increasingly studied and implemented, as demonstrated by their enormous use in market and clinical trials [20,21]. Interestingly, the antisense strategy is also used as a therapeutic novel approach in neurodegenerative disorders in humans [22], according to their excellent results in mouse models. In their paper, Schoch and Miller highlighted the potentiality of ASOs to target specific RNA transcripts (such as SOD1 and C9orf72, which have the recruitment function for Ribonuclease H) thus addressing single-protein malfunctioning that characterizes neurodegenerative diseases. The authors then emphasized that ASOs can be distributed in the Central Nervous System, which is both unforeseen and advantageous in the targeting of synucleinopathies, and concluded that, having seen the preclinical success in SMA and ALS mouse models, ASOs hold the promise for a powerful therapeutic strategy for these challenging diseases [23]. Another important point that suggests that these biomacromolecules are indeed worth deep research is that they can directly target the source of pathogenesis, having a higher chance of success than therapies targeting downstream pathways. Given the promising results, the following six ASOs have been approved to date by the US Food and Drug Administration (FDA) for three neurological disorders: Eteplirsen, Golodirsen, Viltolarsen, and Casimersen for Duchenne muscular dystrophy; Nusinersen for spinal muscular atrophy; and Inotersen for polyneuropathy caused by familial transthyretin amyloidosis [24]. In general, the use of ASOs in therapeutic and research applications has limitations, such as their susceptibility to nuclease degradation, which reduces their efficacy and necessitates repeated administrations [25]; potential toxicity due to off-target interactions [26]; immune response triggering [27]; and decreased tissue accessibility [28].

**Figure 1:**
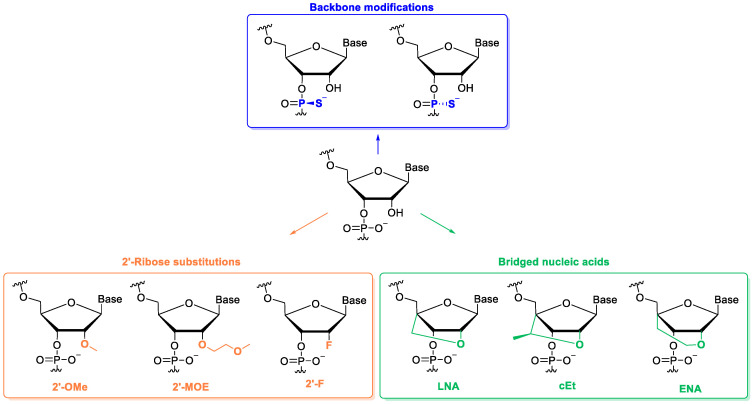
Common chemical modifications to improve classical ASO features.

To enhance delivery, intracellular stability, binding affinity towards the target, and reducing their degradation by nuclease, ASO backbone, and/or sugar moieties are usually modified [20,29]. In this context, common modifications include the following: (i) phosphorothioate backbone incorporation, with the generation of a chiral center and two stereoisomers (Figure 1, blue square); (ii) modification at the 2′ position of the ribose sugar, e.g., introducing 2′-O-methyl, 2′-O-methoxyethyl or 2′-Fluoro moieties (Figure 1, orange square); (iii) or connecting the 2′ and 4′ carbon atoms of the sugar in different ways (Figure 1, green square), as in well-known locked nucleic acids (LNA).

An antisense strategy against PD was recently used by Cole et al., which achieved a 50% reduction in the *SNCA* mRNA levels in rodent αSyn pre-formed fibril (PFF) transmission models of PD, using 17mer 2′-O-methoxyethyl/DNA Gamper ASOs. ASOs were administered by intracerebroventricular injection, preventing pSer129–αSyn aggregate depositions in a dose-dependent manner in the SN and across different brain regions, such as the prefrontal and motor cortex. They also demonstrated that their ASOs can act for a long period after administration (around 160 days per 1 mg single ASO administration), which is an indispensable feature considering that, as well as human conditions, rodent models undergo neurodegeneration in several different central nervous system (CNS) regions after the onset of the pathology. Particularly, PFF rodent models undergo neurodegeneration in different regions of the central nervous system in 4–66 months after PFF injection. Moreover, a specific ASO tested by this group (ASO1) not only prevented the deposition of phosphorylated αSyn but also reduced the number of already established aggregates. This effect was strictly linked to the period that occurred between PFF injection and ASO administration. By administering 0.7 mg of ASO1 after 14 days from PFF injection in mice, they evinced an aggregation reduction of 92% versus the control (PFF only) at day 56. Aggregate resolution of 90% and 51% were highlighted, respectively, at days 60 and 81, following the addition of 1 mg of ASO1 21 days after PFF injection. To verify the effectiveness of this strategy on human *SNCA* transcripts, the authors synthesized two different 20-mer ASOs, following the design and chemistry of already clinically experimented sequences, that they then tested in vitro (on SH-SY5Y human cell lines) and in vivo (αSyn wild type human full-length transgenic mouse). As previously mentioned, these oligonucleotides displayed a dose-dependent and long-term action (lasting 10 days after administration), reducing *SNCA* mRNA levels and αSyn production throughout the brain and spinal cord [30].

**Figure 2:**
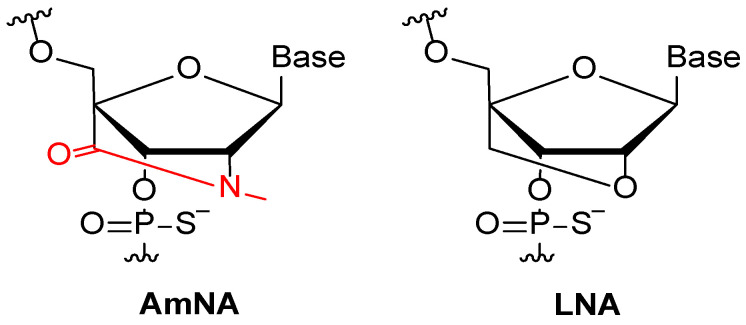
Chemical structure of AmNA and LNA phosphorothioate moieties.

In the same year, Uehara et al. designed and synthesized a family of 50 amido-bridged nucleic acids (AmNA)-modified ASOs (15-mer) to target the coding sequence of *SNCA* mRNA [31]. AmNA-ASOs are analogs of LNA, with an amide bond bridged between the 2′ and 4′ carbon of the ribose (Figure 2) to improve the knockdown efficiency and safety compared to the natural ASOs and LNAs themselves [32].

The authors observed a decrease in *SNCA* mRNA levels of 75.5% in human embryonic kidney 293 cells 24 h after AmNA-ASO transfection. They verified the unique interaction of the best AmNA-ASO of the series (known as ASO^A19^) with αSyn, in comparison with β and γ-synuclein, highlighting the absence of off-target effects. The authors verified the distribution of ASO^A19^ in PD model mice, conjugating it to an Alexa 488 fluorophore. The results suggested the delivery of ASO^A19^ into TH-positive neurons in the SN and the neurons surrounded by TH-positive axons in the striatum. The ASO^A19^ injection, intra-ventricle, efficiently reduced the *SNCA* mRNA levels (52.8%) and expression levels of the αSyn protein (decreasing both soluble and insoluble forms) without the off-targets also in transgenic PD mouse model, TH-*SNCA*-140 m. In addition, the injection of ASO^A19^ improved the motor behavior of mice in direct relationship to the *SNCA* downregulation [31].

However, the ASO features, like a negative charge, high molecular weight, and hydrophilic nature, limit their diffusion across the blood–brain barrier (BBB), reducing efficacy after systemic administration (Table 1). Among the possibilities, intrathecal or intraventricular injections bypass the BBB, directly delivering ASOs to the CNS. Moreover, intranasal administration has recently emerged as a noninvasive method for delivering therapeutic agents to the CNS, utilizing the pathways provided by olfactory and trigeminal nerves [24]. Fascinatingly, Alarcón-Arìs et al. showed that intranasal 2′-O-methyl ASOs conjugated with indatraline (IND), a non-selective monoamine transporter inhibitor [33], reduced αSyn expression in a mouse model of PD [34,35]. First, they demonstrated that 1233-ASO, designed to target *SNCA* mRNA in mouse and human sequences, suppressed αSyn expression in M17-Syn cells (M17 cells overexpressing wild-type human αSyn) by around 60% after 24 h since transfection. By conjugating 1233-ASO to IND moiety, to improve specific ASO cell uptake and internalization, they verified the selective *SNCA* mRNA downregulation in monoaminergic brainstem nuclei. The knockdown of αSyn protein levels in SNc/VTA and in the caudate putamen (CPu) was time-dependent, where 7 days after the intranasal administration, the basal level of αSyn was completely recovered. No DA neurotoxicity was identified such as no change in β and γ-synuclein levels. Moreover, the IND-1233-ASO conjugate efficiently decreased αSyn protein in SNc, and enhanced DA and serotonin neuron neurotransmission in the projection brain areas of the mice, such as CPu [34].To extend this study, the same design was exploited by the authors to disrupt *SNCA* mRNA transcription, uniquely, in the monoamine neurons of rhesus macaques and mice (PD-like mouse model overexpressing human αSyn in DA neurons of SNc/VTA) [35]. The mice were treated with IND-1337-ASO for 28 days through intracerebroventricular application, reaching a maximum decrease in *SNCA* mRNA level 1 day after treatment finalization (mRNA levels reduced to 27% and 43% with 30 µg/day or 100 µg/day therapy, respectively). This result was accompanied by a significant decrease in the human wildtype αSyn protein and its phosphorylated form in SNc and CPu, together [35] with an improvement in the DA neurotransmission in the nigrostriatal pathway. Likewise, IND-1233-ASO was infused in the lateral ventricle of rhesus macaques for 28 days, obtaining a decrease in endogenous αSyn protein levels in the midbrain monoaminergic regions [35].

## 3. Interfering RNAs

Another promising class of molecular tools able to interfere with the translation of specific genes is represented by non-coding RNAs. They are involved in physiological processes and can be used to target “non-druggable” proteins [36]. Indeed, examples of non-coding RNAs employed to treat different pathologies such as cancer [37] have already been described [38]. In this category, microRNAs (miRNAs) [39] and exogenous short-interfering RNAs (siRNAs) [40] are emerging for their post-transcriptional regulation of gene expression [41].

Lin et al. investigated the roles of interactions among αSyn, T199678 (lncRNA), KLF9, and related miRNAs in the PD-related αSyn pathology. In SH-SY5Y cells exposed to αSyn, KLF9 expression was regulated by T199678, whereas T199678 expression was unaffected by KLF9. These results imply that KLF9 is the downstream gene that T199678 regulates, whereas miR-519-3p might be involved as well. Additionally, they verified that the injection of αSyn increased the expression of ROS, which could be suppressed by upregulating T196678, suggesting that T199678 plays an anti-oxidative role in the pathways connected to αSyn. All those findings point to the possibility of an αSyn/T199678/miR-519-3p/KLF9 pathway in PD-associated αSyn pathophysiology [42]. Similar findings were previously reported in 2020, but the authors’ attention was drawn to the role of lncRNA-T199678 in αSyn-induced dopaminergic neuron injury. They showed that overexpressing lncRNA-T199678 reduced αSyn-induced neuron injury by controlling oxidative stress, the cell cycle, and apoptosis. These findings demonstrated that lncRNAs could control the expression of posttranscriptional genes via controlling downstream miRNAs [43].

**Figure 3:**
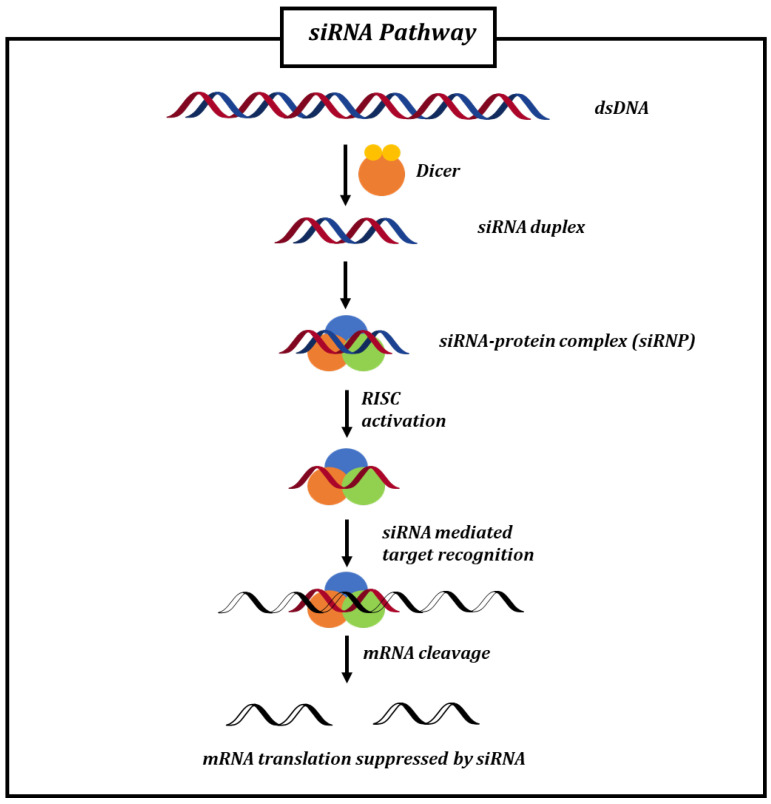
siRNA pathway: ribonuclease protein Dicer recognizes and cleaves DNA double-strand into small fragments (21–23 bp), known as siRNAs, which form the protein RISC complex. Then, siRNA binds the target sequence on mRNA, inducing its cleavage into small fragments (10–11 bp). This results in the suppression of mRNA translation.

However, miRNAs are characterized by low specificity and generally bind multiple targets because of their incomplete base pairing. On the contrary, siRNAs demonstrate higher selectivity by acting on specific mRNA targets. Through extensive research, their mechanism of action has been well characterized (Figure 3). Double-stranded RNA (dsRNA), derived from gene transcription, is cleaved by the enzyme Ribonuclease III into smaller molecules (21–23 base pairs), known as siRNAs. These fragments bind and activate the RNA-inducing Silencing Complex (RISC). Within the complex, one strand of the siRNA, the guide strand, remains associated and targets a specific mRNA sequence, inducing its cleavage into smaller portions. This process leads to the suppression of mRNA translation, highlighting how siRNAs can be effectively utilized to modulate the expression of specific genes. The detailed understanding of the siRNA mechanism has enabled the development of more efficient and effective siRNAs. Indeed, by tailoring the guide strand for mRNA binding, siRNAs’ high specificity reduces off-target effects, resulting in robust and persistent gene suppression. The ability to specifically target disease-causing genes has broadened the range of therapeutic applications for siRNAs, which have found application even in the treatment of neurodegenerative diseases, including PD, to regulate the translation of αSyn and reduce the toxic effects caused by its accumulation.

One of the first examples reported in this field was the work of Gorbatyuk et al., which used αSyn siRNA to downregulate *SNCA* expression [44]. To facilitate their transport to the target, adeno-associated virus (AAD) [45] has been used as the vector for the delivery of two different siRNAs (siRNA-312 and siRNA-163). These two sequences led, respectively, to 93% and 60% knockdown efficiency in vitro, after injection in HEK-293 cells. Promising results have also been obtained with experiments in vivo, where 4 × 10^9^ genomes containing virus particles were injected in the SNc of rats and, 4 weeks after administration, *SNCA* mRNA was reduced to 10% with siRNA-163 and to 5% with siRNA-312. Nevertheless, despite these encouraging achievements, authors observed significant toxic effects; in particular, simultaneous with the *SNCA* mRNA knockdown, the tested siRNAs did not alter TH expression but increased TH phosphorylation and decreased DA levels, resulting in negative consequences for the CNS.

Toxic effects generated by this approach have also been reported in the work of Zharikov et al., who observed that, at the physiological level, a reduction in αSyn of above 70% is tolerated for up to 12 months in mice; after this period, degeneration of the organisms’ CNS has been observed [46]. On the contrary, Di Monte et al. obtained more promising results after they had designed and synthesized a specific 21 bp siRNA sequence to target *SNCA* transcripts in monkey brains. They analyzed both mRNA and protein expression, by polymerase chain reaction and immunohistochemistry, in different regions of the brain, finding that administration of 5.4 µg/h, over four weeks, led to a 50% reduction in *SNCA* mRNAs levels in the anterior and posterior SN. To confirm the efficacy of this system, its toxicity on animals has been assessed, where the selected siRNAs affected neither the number nor the phenotype of the TH-immunoreactive neurons. Furthermore, this kind of treatment did not reduce the production of DA and its metabolites [47]. Fascinatingly, Zharikov et al. designed two promising siRNAs targeting the *SNCA* mRNA that permitted a knockdown of 75% (siRNA270) and 85% (siRNA526) in the αSyn expression of CHO cells expressing rat αSyn. Due to the promising in vitro results, the authors synthesized an shRNA corresponding to the most effective siRNA studied (siRNA526) and exploited the adeno-associated virus serotype 2 (AAV2) vector for in vivo studies. After three weeks of AAV–sh[*SNCA*] infusion into the substantia nigra dopaminergic neurons of rats, the authors recorded a 35% reduction in αSyn expression. Using a rotenone model of PD, which mimics the selective neurodegeneration seen in PD despite widespread mitochondrial inhibition, the authors demonstrated that αSyn knockdown reduced its accumulation, by protecting motor function and preserving striatal dopaminergic terminals, nigral neurons, and dendrites. Additionally, this study observed a 20% reduction in striatal dopamine levels without motor deficits or neurodegeneration, suggesting intact dopaminergic terminals. These findings implied that αSyn may affect dopamine handling and storage, and its knockdown might protect against dopamine-induced mitochondrial damage. Given the safety of AAV2 vectors in human trials, targeting αSyn with shRNA holds significant translational potential as a neuroprotective strategy in PD and warrants further investigation [48].

Following the same aim reported for 1233-ASO, Alarcón-Arìs et al. conjugated an IND moiety to a siRNA (499-siRNA), which encompassed the same *SNCA* gene sequence of 1233-ASO [34]. Despite IND-499-siRNA significantly and selectively reducing the *SNCA* mRNA levels in treated mice 1 day after the intranasal administration, the complete recovery of αSyn expression was obtained after only 3 days (IND-1233-ASO effect was maintained for 72 h), resulting in a less effective outcome than IND-1233-ASO [34]. The worse efficiency displayed by siRNAs can be attributed to several reasons, such as their higher molecular weight and the possibility of hindering the formation of RISC and interfering with other important physiological processes. This class of non-coding RNAs presented also other drawbacks, like low stability in the physiological environment, poor cellular uptake, and low permeability to the BBB). Therefore, during the last few years, novel delivery systems, such as nanoparticles, lipid-based systems, and polymeric materials have been developed to overcome these limitations [49].

Richter et al. exploited siRNAs to downregulate the *SNCA* expression and, to improve the efficiency of their method, they used a positively charged polymer, Polyethyleneimine, to deliver their oligonucleotide to the desired target [50]. This complex displayed low toxicity on SH-SY5Y cells (22.5% at 150 pmol of siRNA) and was efficiently distributed around ventricles and neurons. The authors determined the effect of their system in vivo, in Thy1-αSyn mice. The *SNCA*/mRNA levels were analyzed through qRT-PCR and a dose of 0.75 µg complex was found to cause 67% of the translation inhibition. Immunohistochemistry revealed that the same amount of siRNA induced a 50% decrease in αSyn protein levels, in both hemispheres in the striatum, cortex, and medial septum. Therefore, this represents a promising example of the modulation of gene expression with short RNA sequences, demonstrating its efficacy even in vivo, with the proper delivery system [50].

To increase permeability through the BBB, Rissman et al., with an analog approach, designed a peptide based on Apolipoprotein B (ApoB) to facilitate the transport of oligonucleotides to the brain, exploiting a 38-amino acid receptor-binding domain [51]. To verify the efficiency of the delivery system, authors incubated N2A mouse cholinergic neuronal cells with a complex constituted by ApoB, the siRNA (siRNAαSyn), and a fluorescent probe, fluorescein isothiocyanate (FITC). The system, ApoB/siRNAαSyn–FITC, was distributed in the cytoplasm after 3 h, reaching the maximum intensity after 24 h. Subsequently, the effect on the modulation of αSyn levels was evaluated as follows: N2A cells were treated with 100 pmol of ApoB/siRNAαSyn, and protein levels were measured through immunocytochemistry and immunoblot assay. After 24–48 h, a 30% reduction in the αSyn protein levels was observed. The ability of ApoB/siRNAαSyn to reach the desired target has been determined with co-localization experiments using the αSyn1-antibody, confirming that this system can penetrate BBB and is distributed in neurons. αSyn modulation has been investigated also in vivo, in αSyn tg mice, using the αSyn-antibody. In mice presenting a high accumulation of αSyn in the neuropil and neuronal cell bodies in the neocortex, hippocampus, and striatum, treatment with ApoB/siRNAαSyn caused a 65–70% reduction in αSyn in the neocortex, 40–50% in the hippocampus, and 50–55% in the striatum [51]. These experiments were particularly meaningful because demonstrated that siRNA successfully reduced the number of protein aggregates in mice.

Similarly, a more recent work by Leitão et al. demonstrated that the conjugation of a 2′-OMe-ASO to a peptide fragment of Apolipoprotein B (ApoB), useful for the systemic transport of oligonucleotides to the CNS, could be an efficient approach for lowering αSyn in a mouse model [52]. They postulated that the systemic administration of modified ApoB^11^/2′-OMe-ASO would efficiently cross the BBB and reduce αSyn expression in the AD brain thus enhancing neuronal survival and memory. Their conjugate was administered intraperitoneally to AD mice at a concentration of 2 mg/kg at different months of age (6, 7, and 8), demonstrating a significant reduction in phosphorylated αSyn in all regions of the hippocampus of AD mice injected with ApoB^11^/2′-OMe-ASO compared to a control experiment, by immunohistochemical experiments. Additionally, intraperitoneal injections of ApoB^11^/2′-OMe-ASO in AD mice showed a noteworthy reduction in the number of Aβ plaques, both within the cortex and the hippocampus [52].

The strategy of using conjugated ASOs to cross the BBB was used in another work and, in principle, could be applied for the reduction in αSyn levels in the brain. Wang et al. explored the potential of small apoptotic bodies (sABs) from brain metastatic cancer cells for brain-targeting drug delivery. It was found that anti-TNF-α ASO combined with cationic konjac glucomannan can be successfully loaded into sABs and have extraordinarily high brain delivery efficiency. Their study pointed out that ASO-loaded sABs are transcytosed by brain microvascular endothelial cells to penetrate the BBB and are eventually taken up by microglial cells in the brain. As a further improvement, this strategy could be applied to specific ASOs that can knock down αSyn levels in the AD brain [53] (Table 2).

## 4. CRISPR-Mediated Alternative Mechanisms

CRISPR–Cas-based gene editing stands out for its ability to introduce heritable genome modifications using short guide RNAs, making it an extremely successful gene therapy method [54]. Among the CRISPR–Cas systems, CRISPR/Cas9 is a versatile, programmable tool for creating DNA double-strand breaks in various organisms and cell types, ranging from bacteria to human pluripotent stem cells, allowing for the treatment of human diseases [55]. Specifically, it has shown encouraging effects in treating neurodegenerative diseases, including PD.

In 2018, for example, Kantor et al. developed an all-in-one lentiviral vector employing CRISPR-deactivated Cas9 (dCas9) and DNA-methyltransferase 3A (DNMT3A) to edit the *SNCA* intron 1 methylation levels in human induced pluripotent stem cell (hiPSC)-derived dopaminergic neurons from a PD patient with the *SNCA* triplication locus (*SNCA*-Tri). Given that the differential methylation of the CpG-rich regions was evidenced in the *SNCA* intron 1 of PD brains, the authors developed four distinct guide RNAs (gRNAs), highlighting that a reduction in the *SNCA* mRNA levels was achieved exclusively by targeting the 3′-end of the CpG island region (with gRNA4). The lentiviral vector carrying the gRNA4–dCas9–DNMT3A combination showed low off-target effects on global DNA methylation when compared to overexpressed untargeted DNMT3A, emphasizing the importance of gRNA in mitigating off-target risks. Additionally, the authors verified that this approach resulted in the effective downregulation of both *SNCA* mRNA (around 30%) and αSyn protein expression (around 25%) in *SNCA*-Tri hiPSC-derived neurons, with a restoration of disease-related phenotypic perturbations [56].

Chen et al. used CRISPR/Cas9n editing to show that deleting alleles of the *SNCA* gene in human embryonic stem cells (hESCs) and induced pluripotent stem cells (iPSCs) reduced or eliminated αSyn expression from mDA neurons and conferred resistance to the αSyn PFF-induced formation of Lewy-like pathology. hESCs were cloned with a complete deletion of the *SNCA* exon 2, giving rise to the RC17-hESC parental line to produce a number of *SNCA*^+/−^ and *SNCA*^−/−^ clonal cell lines. Synucleinopathy was induced in mDA neurons by the administration of PFFs, where the neurons were then maturated and immunostained for β-III tubulin and pSer129-αSyn, a biomarker of aggregated αSyn. In their experiments, they observed very few or no pSer129-αSyn structures in *SNCA*^+/−^ and *SNCA*^−/−^ mDA neurons, and the exposure of the same entities to equimolar monomeric αSyn was unable to produce pSer129-αSyn structures. Their data suggest that the loss of αSyn induced by the allelic modification of the *SNCA* gene in their models confers a total resistance to the experimentally induced synucleinopathy [57].

CRISPR–Cas9 can be also employed to delete mutations, offering a promising strategy for repairing genetic abnormalities linked with a variety of disorders. In this regard, the missense mutation Ala53Thr (A53T) in *SNCA*, which has been identified as one of the most significant risk factors in early-onset PD, is an intriguing target [58]. Interestingly, Chen et al. investigated the neurons generated from hiPSCs from PD patients with A53T and *SNCA*-triplication mutations, as well as their CRISPR-edited counterparts, and discovered that the absence of *SNCA* gave resistance to Lewy pathology [59]. In support of these results, in 2022, Yoon et al. demonstrated that using the CRISPR–Cas9 system to delete the A53T-*SNCA* gene mutation improved PD-related conditions, including αSyn overproduction, reactive microgliosis, dopaminergic neurodegeneration, and motor symptoms [60].

## 5. Small Molecules

Antisense strategies have been widely employed to target RNA sequences; however, this approach presents the following non-negligible limitations: in particular, they are often characterized by poor stability and high charge density, which complicates their delivery to the target [61]. Therefore, developing small molecules with easily tunable structural features that can interfere with RNA transcription represents a promising alternative. In the last decades, the discovery of RNA riboswitch regulatory elements that bind small molecule metabolites has reinforced this idea [62]. However, despite the rising number of small molecules targeting nucleic acid sequences, their application on *SNCA* mRNA remains limited. Due to the lack of small molecules for treating neurodegenerative diseases, discovering novel drugs is particularly urgent. One promising approach to accelerate this process is high-throughput screening techniques, which exploit libraries of existing compounds that can be tested on the target of interest [63].

**Figure 4:**
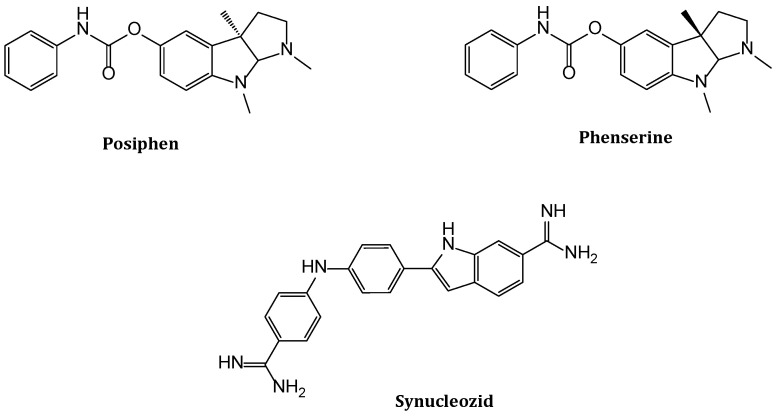
Chemical structures of ligands targeting 5′-UTR of *SNCA* mRNA.

Rogers et al. exploited this approach and screened a library of 720 natural compounds to individuate molecules able to regulate *SNCA* translation upon binding with the stem loop located on its 5′-untranslated region (UTR) [64]. Through a luciferase assay, they tested different classes of products as potential *SNCA* regulators, in concentrations between 0.08 µM and 10 µM; the most efficient class of inhibitors were the plant glycosides, such as Strophathidine, that were able to reduce *SNCA* expression in SK-N-SH and SH-SY5Y cells (65% reduction), with low toxicity (IC_50_ > 10 µM). Also, Mycophenolic acid, Posiphen, and (−)-Phenserine showed a significant inhibition of *SNCA* translation and expression (Figure 4) [64].

In subsequent work, Rogers et al. focused their research on a cholinesterase inhibitor, Phenserine, and its non-cholinergic enantiomer, Posiphen, to modulate the expression of the *SNCA* gene [65]. It has been demonstrated that these two compounds can reduce amyloid precursor protein (APP) levels in the treatment of AD, through the binding of 5′-UTR mRNA. Due to the similarity of 5′-UTR of APP with *SNCA*, authors speculated that they could also represent a promising therapeutic approach against PD. Indeed, through luciferase assay, it was verified that Posiphen was an efficient inhibitor of *SNCA* in H2A neural cells (IC_50_ = 10 µM), while Phenserine only had a negligible effect. The ability of Posiphen to downregulate *SNCA* translation was also evaluated on primary neurons from wild-type mice and PAC *SNCA* transgenic mice, causing a 75% reduction at 1 µM. Because of these interesting results, the same authors studied the effect of this molecule on αSyn levels in mice brains and its effects on the gastrointestinal system [66]. Mice treated with Posiphen (3 mg/kg and 10 mg/kg) recovered their colon motility, compared to untreated *hSNCA* individuals, and this effect lasted for several months after the end of the treatment. Furthermore, the authors analyzed the levels of αSyn in mice brains at different concentrations of Posiphen, after a period of incubation of 21 days. Analyzing the extracts through a Western blot and an ELISA assay, the highest reduction (28%) in the brain was achieved at 65 mg/kg. These experiments confirmed the positive effects of Posiphen in vivo, proving its efficacy as a potential novel drug for the treatment of PD [66].

Recently, Zhang et al. described the design of a novel compound, Synucleozid, to specifically target the Iron-Responsive Element (IRE), located in the 5′-UTR of *SNCA* mRNA, responsible for its translation [67]. The design of a molecule with high affinity and selectivity for a specific nucleic acid sequence is a hard challenge; for this aim, authors exploited their sequence-based design, Inforna [68], to identify different potential ligands. Molecules provided by the database were chosen for their ability to recognize two specific structural elements, typical of IRE, such as one-nucleotide internal loop and one-nucleotide bulge with GC and GU closing base-pairs. Ligands were tested in SH-SY5Y cells and their ability to reduce αSyn expression was evaluated. Synucleozid gave the best outcome with IC_50_ = 500 nM. To confirm that its activity was due to the interaction with IRE, the authors demonstrated that this ligand inhibited only translation and not transcription. Subsequently, to evaluate the selectivity of Synucleozid for IRE on *SNCA* mRNA, they tested its ability to suppress the translation of other IRE-containing mRNAs, such as APP, prion protein, and ferritin, and they found that it does not affect these targets. The ligand’s affinity for the IRE binding site was investigated in vitro by replacing the bulge with fluorescent 2-Aminopurine; the emission intensity variation following engagement with Synucleozid demonstrated that the molecule interacted with this specific site. Furthermore, exploiting the ASO-binding map in three different assays, they proved the Synucleozid association with the bulge. Moreover, the selectivity of Synucleozid in the cellular environment was determined through transcriptome and protein studies on SH-SY5Y cells treated with the ligand and an αSyn small-interfering siRNA. These studies showed that ligand and siRNA have 53 target proteins in common, suggesting a similar mechanism of action.

Four years later, the same group expanded their study on Synucleozid. In their latest work, they used several synergistic approaches to identify new RNA-targeting small molecules with favorable drug-like physicochemical properties. Using a novel screening method called AbsorbArray, they easily identified compounds that bind to the *SNCA*–IRE. This led to the discovery of a new compound, Synucleozid-2.0, which exhibited improved potency, selectivity, and drug-like properties compared to the original Synucleozid.

They discovered that Synucleozid-2.0 inhibited *SNCA* translation in SH-SY5Y cells dose-dependently, with an IC_50_ of approximately 2 μM. To assess its specificity, the researchers tested its effects on other mRNAs with IREs in their untranslated regions, such as ferritin and APP, which have IREs in their 5′-UTRs, and the transferrin receptor, which has an IRE in its 3′-UTR. Notably, these IREs have no structural overlap with the *SNCA* IRE, and Synucleozid-2.0 did not affect their protein levels. Interestingly, Synucleozid-1.0, while potently decreasing α-Syn levels, also had a modest effect on ferritin. To further investigate the selectivity of Synucleozid-2.0, they conducted competition binding experiments, using a series of 12 unlabeled RNAs with mutations introduced into the IRE, particularly to create A, G, or U bulges. Only RNA-1, with an A bulge mutated to an AU pair, significantly reduced Synucleozid-2.0 binding by 12-fold, whereas binding remained unchanged with other competitor RNAs. This suggests that Synucleozid-2.0 specifically binds to the A bulge and its surrounding base pairs in the *SNCA* IRE, interacting with the three-dimensional structure of the RNA [69]. In conclusion, these works proved the strong effect of small molecules, Synucleozid and Synucleozid 2.0, on αSyn expression, demonstrating that their activity can be mainly attributed to the specific binding of the IRE region on *SNCA* mRNA [67].

Nowadays, examples of small molecules used for PD treatment are still limited; however, the positive results described in the previous examples will encourage the optimization of structural properties of novel molecules to achieve high selectivity and improved activity, resulting in the discovery of innovative drugs for this pathology (Table 3).

## 6. Nucleic Acid Secondary Structures as Pioneering Potential Therapeutic Targets

**Figure 5:**
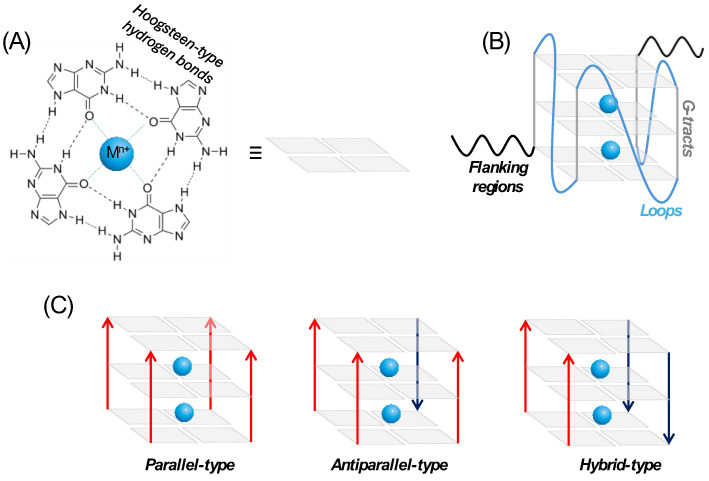
Schematic representation of (**A**) square-planar G-tetrad; (**B**) backbone of the intramolecular G-quadruplex structure; (**C**) G4 topologies: parallel-, antiparallel-, and hybrid-type G4 structures.

A groundbreaking approach in medicinal chemistry is the targeting of nucleic acid secondary structures, among which G-quadruplex (G4) motifs are the most studied. G4s are extremely stable DNA or RNA non-canonical supramolecular structures formed by guanine-rich sequences [70]. The building blocks of any G4 are planar quartets, known as G-tetrads, constituted of four guanines connected via Hoogsteen base-pairing (Figure 5A). Two or three G-tetrads can stack on top of each other, leading to the formation of G4s backbone (Figure 5B) [71]. The presence of specific monovalent metal cations in the central channel formed by the stacked G-quartets affects the stability of the G4s in the following order: K^+^ >> NH_4_^+^ ≥ Na^+^ >> Li^+^ [72]. DNA G4s are highly polymorphic structures and present different topologies depending on the orientation of the strands and loop configurations. Specifically, the parallel type is when all four strands run in the same direction (either all 5′ to 3′ or all 3′ to 5′); the antiparallel type is when two strands run in one direction (5′ to 3′) and the other two run in the opposite direction (3′ to 5′); and the hybrid type is when three strands run in one direction and the fourth strand runs in the opposite direction (Figure 5C) [71]. While DNA G4s can adopt different topologies, the presence of uracil instead of thymine and ribose sugar instead of deoxyribose allow for only a parallel RNA-G4 topology folding [72]. Moreover, these features led to enhanced intramolecular interactions and stability within RNA-G4s, as demonstrated by higher thermodynamic stability compared with their DNA counterparts [73,74].

**Figure 6:**
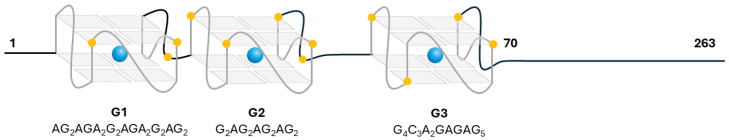
Cartoon representing the three G4 motifs identified in the 5′-UTR mRNA region of the *SNCA* gene by Koukouraki et al. [78], together with all the G to A mutations tested (highlighted in yellow).

G-quadruplexes are not randomly distributed across the cell genome but are mainly clustered in “hot” genomic regions, such as telomers or gene promoters, open reading frames, and untranslated regions. Consequently, their involvement in biological processes such as the regulation of gene expression, genomic instability, telomerase dysfunction, and viral transcription, has made G4s an interesting target in medicinal chemistry [75,76]. About 3000 human genes are known to contain potential G4-forming sequences in the 5′-UTR and the 3′-UTR, and RNA G4s have been shown to play important regulatory roles in enhancing or inhibiting the translation of various proteins associated with a range of neurological diseases, including AD, PD, and fragile X syndrome (FXS) [77]. Considering that the human *SNCA* mRNAs possess a long 5′-UTR (264 nucleotides) with a high content of GC bases (66%), Koukouraki et al. evaluated the propensity of this region in folding into RNA G4s. Bioinformatic analysis revealed the presence of three non-overlapping G4 motifs (G1, G2, and G3 in Figure 6), which operate as negative regulators of translation. This behavior was demonstrated by the transfection in HEK-293 cells of four psiCHECK2 reporter constructs, in which each of the predicted G4 motifs or all together were mutagenized (G to A mutations, Figure 6).

Mutations in the G1, G2, and G3 motifs and a combined G1/G2/G3 mutated sequence resulted in increased Renilla luciferase activity than the wild-type *SNCA* 5′-UTR construct of about 30%, 29%, 36%, and 45%, respectively. This suggested that these G4 motifs negatively regulate translation when intact. The differential effects observed among the motifs suggest distinct mechanisms of action. For instance, while G1 and G2 seem to influence mRNA stability or transcription, G3 appears to directly affect the translation machinery [78]. Although this work has no experimental evidence of G4 formation, it represents the first proof that if these G4 motifs are preserved in native *SNCA* mRNA, they are likely to play an important role in controlling *SNCA* expression. In 2024, Pirota et al. proposed a novel strategy for lowering αSyn through the downregulation of *SNCA* by targeting different G-quadruplex structures. As mentioned, the folding modulation of G4s is an emerging strategy to modulate translation and transcription processes They identified two G4 motifs—one straddles the transcription start site of the *SNCA* gene (p*SNCA*) and one in the 5′-UTR of *SNCA* mRNA spanning the G1 and G2 sequences identified by Koukouraki et al. (m*SNCA*) [78]. Both motifs were confirmed to fold into stable G4s. Stabilizing p*SNCA* G4 by well-known G4-ligands, the authors verified an increase in the *SNCA* mRNA in differentiated SHSY5Y cells, suggesting a boosting role in the *SNCA* transcription of p*SNCA* G4. Additionally, they recorded a reduction of 50% in αSyn production deriving from m*SNCA* stabilization, reinforcing the results of Koukouraki et al. Much more intriguing was the result achieved with a peptide nucleic acid sequence (NLS-PNA-C343) designed to hybridize with the nucleobases of the p*SNCA* sequence selectively. The treatment of differentiated SHSY5Y cells with NLS-PNA-C343 resulted in a 70% drop in mRNA and protein levels, demonstrating that the interaction with p*SNCA* can successfully influence gene and protein expression [79].

Although G4 targeting has not yet been validated as a treatment, ongoing clinical trials on stabilizing G4 ligands (e.g., Pidnarulex and SOP1812) [80,81] highlight the promise of targeting these structures. Furthermore, G4s have a significant advantage as targets because of their well-defined folded structures, which allow for the development of possible active agents like pharmacological targeting of folded proteins but at the DNA and RNA level. This cutting-edge method has the potential to transform therapeutics by precisely controlling gene expression and protein production, paving the path for innovative, truly viable therapeutic options in the future.

## 7. Conclusions

The different approaches reported demonstrate that the downregulation of the *SNCA* gene by nucleic acid-targeting may reduce αSyn content slowing down/stopping PD pathogenesis.

It is important to note that the ability to specifically downregulate a gene, while limiting off-targets, is an essential feature to remove non-specific activities and severe collateral effects that may oppose the potential therapeutic effect.

In this sense, ASOs provide a powerful tool for gene silencing with high specificity, guaranteed by hybridization with complementary strands also in a complex physiological environment, particularly valuable for the knockdown of αSyn production. Despite significant advancements and clinical successes, several challenges remain, including stability, off-target effects, immune responses, and efficient delivery to target tissues. Ongoing research into chemical modifications and novel delivery methods continues to improve the therapeutic potential of ASOs, particularly for CNS disorders where traditional delivery routes are inadequate. The promising results in preclinical and clinical studies underscore the potential of ASOs to revolutionize treatments for genetic and neurodegenerative diseases. In Parkinson’s disease models, specific ASOs have shown significant reductions in *SNCA* mRNA and αSyn protein levels, improved motor behavior, and long-term action after administration. Innovative delivery methods, such as intrathecal, intraventricular, and intranasal administration, have been developed to bypass the blood–brain barrier and enhance CNS delivery. Notably, intranasal ASOs conjugated with indatraline demonstrated effective αSyn knockdown in PD models, offering a non-invasive therapeutic option.

In parallel, non-coding RNAs, including microRNAs and exogenous short-interfering RNAs, are emerging as promising molecular tools in treating Parkinson’s disease. While miRNAs typically bind multiple targets due to incomplete base pairing, siRNAs exhibit higher selectivity by targeting specific mRNA sequences, leading to highly selective gene silencing. This high specificity makes siRNAs more valuable for therapeutic applications. Among the most promising results in modulating αSyn expression in PD models, recent advancements have focused on improving siRNA delivery systems to overcome challenges such as low stability, poor cellular uptake, and limited blood–brain barrier permeability. Examples include using polyethyleneimine polymers, ApoB-based peptides, and small apoptotic bodies to enhance siRNA delivery and efficacy in the brain. Despite some challenges, ongoing research into improved delivery systems and optimization techniques continues to enhance the viability and effectiveness of siRNA-based therapies. These advancements promise to unlock new avenues for treating various genetic and protein-related disorders, offering hope for more targeted and efficient therapeutic strategies.

CRISPR–Cas-based gene editing has emerged as a promising method for introducing heritable genome modifications, using guide RNAs to target specific genes. Among these systems, CRISPR/Cas9 has been particularly effective in creating DNA double-strand breaks across a wide range of organisms and cell types, including human pluripotent stem cells, making it a valuable tool for treating various diseases, including neurodegenerative disorders like PD. By targeting specific regulatory regions or genetic mutations, CRISPR-based interventions can reduce pathological αSyn protein levels and protect neurons from disease-related damage, offering a promising avenue for future therapies. The success of these approaches in preclinical models suggests that they could eventually translate into effective treatments for patients.

An alternative to antisense strategies and CRISPR-mediated approaches is the development of small molecules. While examples of small molecules for PD treatment are still limited, the promising results in inhibiting αSyn expression highlight their potential as a solid starting point for developing more efficient and specific therapies. Optimizing the structural properties of new molecules could lead to high selectivity and improved activity, paving the way for innovative drugs to treat PD. The success of compounds like Synucleozid and Synucleozid-2.0 demonstrates the feasibility of targeting specific RNA elements such as IRE to regulate *SNCA* gene expression, effectively reducing αSyn production without affecting other IRE-containing mRNAs. This approach offers a promising avenue for future therapeutic development.

In addition, the pioneering targeting of G-quadruplexes within the *SNCA* gene represents an innovative approach to regulating gene expression and αSyn protein production. Despite not yet being validated as a treatment, ongoing clinical trials with G4-stabilizing ligands highlight the potential of this approach. The well-defined structures of G4s make them advantageous targets for drug development, akin to targeting folded proteins. This innovative method could transform therapeutics, offering precise control over gene expression and paving the way for new, effective treatments.

## Figures and Tables

**Table 1:** Summary of the ASO-based biomolecules and their effect on the target in the biological models examined in this chapter.

Entity	Biological Model	Effect on the Target	Reference
17-mer 2′-O-methoxyethyl/DNA Gamper ASO	Rodent αSyn pre-formed fibril	50% reduction in *SNCA* mRNA levels.	Cole et al. [30]
	SH-SY5Y human cell	Reduction in *SNCA* mRNA levels and αSyn production.	Cole et al. [30]
ASO^A19^	PD mouse model TH-*SNCA*-140 m	Decrease in *SNCA* mRNA levels of 52.8% and αSyn production.	Uehara et al. [31]
	Human embryonic kidney 293 cells	Decrease in *SNCA* mRNA levels by 75.5%.	Uehara et al. [31]
1233-ASO	M17-Syn cells	60% suppression of αSyn expression.	Alarcón-Arìs et al. [35]
IND-1233-ASO	Rhesus macaques	*SNCA* mRNA transcription disruption.	Alarcón-Arìs et al. [35]
	PD-like mouse model overexpressing human αSyn in DA neurons of SNc/VTA	*SNCA* mRNA levels were reduced to 27% and 43% with 30 µg/day or 100 µg/day therapy respectively.	Alarcón-Arìs et al. [35]
		Decrease in human wildtype αSyn protein.	Alarcón-Arìs et al. [35]

**Table 2:** Summary of the short-interfering RNAs and their effect on the target in the biological models examined in this chapter.

Entity	Biological Model	Effect on the Target	Reference
siRNA-312	HEK-293 cells	93% *SNCA* knockdown	Gorbatyuk et al. [44]
siRNA-163	HEK-293 cells	60% *SNCA* knockdown	Gorbatyuk et al. [44]
siRNA-270	Rat CHO αSyn cells	75% αSyn expression knockdown	Zharikov et al. [48]
siRNA-526	Rat CHO αSyn cells	85% αSyn expression knockdown	Zharikov et al. [48]
AAV-sh[*SNCA*]	substantia nigra dopaminergic neurons of rats	35% reduction in αSyn expression	Zharikov et al. [48]
PEI-siRNA	Thy1-αSyn mice	67% *Snca* mRNA translation inhibition 50% decrease in αSyn expression	Richter et al. [50]
ApoB/siRNAαSyn-FITC	N2A mouse cholinergic neuronal cells	30% reduction in αSyn protein levels 70% reduction in αSyn in the neocortex, 40–50% in the hippocampus and 50–55% in the striatum	Rissman et al. [51]
ApoB^11^:2′-OMe-ASO	AD mice	significant reduction in phosphorylated αSyn in all regions of the hippocampus	Leitão et al. [52]

**Table 3:** Summary of the small molecules and their effect on the target in the biological models examined in this chapter.

Entity	Biological Model	Effect on the Target	Reference
Strophathidine	SK-N-SH and SH-SY5Y cells	65% reduction in *SNCA* expression IC_50_ > 10 µM	Rogers et al. [64]
Posiphen	H2A neural cells	*SNCA* inhibition IC_50_ = 10 µM	Mikkilineni et al. [65]
	primary neurons from wild-type mice and PAC *SNCA* transgenic mice	75% reduction in *SNCA* translation	Mikkilineni et al. [65]
Synucleozid	SH-SY5Y cells	Reduction in αSyn expression IC_50_ = 500 nM	Zhang et al. [67]
Synucleozid 2.0	SH-SY5Y cells	Dose-dependent inhibition of *SNCA* translation IC_50_~2 μM	Tong et al. [69]
